# Experimental repetitive mild traumatic brain injury induces deficits in trabecular bone microarchitecture and strength in mice

**DOI:** 10.1038/boneres.2017.42

**Published:** 2017-12-19

**Authors:** Chandrasekhar Kesavan, Nikita M Bajwa, Heather Watt, Subburaman Mohan

**Affiliations:** 1 Musculoskeletal Disease Center, VA Loma Linda Healthcare System, Loma Linda, CA, USA; 2 Department of Medicine, Loma Linda, CA, USA; 3 Department of Orthopedic Surgery, Loma Linda University, Loma Linda, CA, USA

## Abstract

To evaluate the long-term consequence of repetitive mild traumatic brain injury (mTBI) on bone, mTBI was induced in 10-week-old female C57BL/6J mice using a weight drop model, once per day for 4 consecutive days at different drop heights (0.5, 1 and 1.5 m) and the skeletal phenotype was evaluated at different time points after the impact. *In vivo* micro-CT (μ-CT) analysis of the tibial metaphysis at 2, 8 and 12 weeks after the impact revealed a 5%–32% reduction in trabecular bone mass. Histomorphometric analyses showed a reduced bone formation rate in the secondary spongiosa of 1.5 m impacted mice at 12 weeks post impact. Apparent modulus (bone strength), was reduced by 30% (*P*<0.05) at the proximal tibial metaphysis in the 1.5 m drop height group at 2 and 8 weeks post impact. *Ex vivo* μ-CT analysis of the fifth lumbar vertebra revealed a significant reduction in trabecular bone mass at 12 weeks of age in all three drop height groups. Serum levels of osteocalcin were decreased by 22%, 15%, and 19% in the 0.5, 1.0 and 1.5 m drop height groups, respectively, at 2 weeks post impact. Serum IGF-I levels were reduced by 18%–32% in mTBI mice compared to contro1 mice at 2 weeks post impact. Serum osteocalcin and IGF-I levels correlated with trabecular BV/TV (*r*^*2*^=0.14 and 0.16, *P*<0.05). In conclusion, repetitive mTBI exerts significant negative effects on the trabecular bone microarchitecture and bone mechanical properties by influencing osteoblast function via reduced endocrine IGF-I actions.

## Introduction

Mild traumatic brain injury (mTBI) is an injury that occurs as a result of an external force, typically caused by falls, accidents, sports injuries, and military conflicts.^1^ Many individuals diagnosed with mTBI are left with significant long-term neurological and physical impairments that have a major catabolic effect on other parts of the body, known as polytrauma. Data from the Department of Defense revealed that ~235 046 service members who served in the Army, Air Force, Navy, and Marines have been diagnosed with a TBI; however, these numbers do not account for individuals that have not reported an injury or received medical care. Furthermore, the Center for Disease Control and Prevention reported that 2.5 million people in US have sustained a TBI.^1^ As the number of people with mTBI increases annually, the long-term disabilities and medical costs are likely to rise concurrently. Therefore, identifying strategies that may reduce the rate of TBI mediated secondary conditions may improve an individual’s quality of life and associated healthcare and disability costs.

In healthy individuals, the hypothalamus transmits signals from the brain to the pituitary gland by stimulating endocrine factors that are essential for maintaining homeostasis. In TBI, the hypothalamus–pituitary axis is disrupted, causing a reduction in endocrine factors produced from the pituitary gland,^2–8^ leading to long-term adverse effects on target tissues. A 12 month prospective study found that the rate of post TBI hypopituitarism was 33% and 23% at 3 and 12 months, respectively.^9,10^ A second clinical study reported anterior pituitary dysfunction in 56% and 36% of TBI patients at 3 and 12 months, respectively.^11^ A recent study has found that 70% of patients with TBI have hypothalamic-pituitary dysfunction.^12^ Overall these data demonstrate that hypothalamus–pituitary dysfunction is an early and key feature of TBI.

Hypothalamus–pituitary dysfunction results in multiple hormone deficiencies, with growth hormone (GH) deficiency being the most commonly diagnosed in patients with mild, moderate, or severe TBI. Kelly *et al*.^13^ reported that approximately 18% of TBI patients developed GH deficiency. Similarly, Aimaretti *et al*.,^9^ showed that 21% and 20% of TBI patients were deficient in GH or insulin-like growth factor-I (IGF-I) levels, respectively. Likewise, Lieberman *et al*.^14^ reported GH deficiency in 14.6% of TBI patients. Growth hormone is a well-known regulator of skeletal homeostasis and is critically involved in regulating normal longitudinal bone growth and bone mass.^15,16^ Clinical studies have shown that adults deficient in GH suffer from low bone turnover osteoporosis, which leads to an increased risk of fracture and mortality.^17,18^ Since patients examined with varying severities of TBI (mild, moderate, and severe) have shown reduced GH levels, we hypothesized that decreased GH levels induced by mTBI exerts a negative effect on bone mass and strength over time. To test this hypothesis, we induced mTBI in C57BL/6J female mice experimentally using a well-established weight drop model and evaluated the consequences of varying impacts on bone mass, strength, and serum markers of bone metabolism over a period of 12 weeks.

## Materials and methods

### Animals

Nine week old female C57BL/6J mice (*n*=10/group) were purchased from Jackson Laboratory (Bar Harbor, ME, USA). All the animals were housed under standard conditions with 14 h of light and 10 h of darkness, with unrestricted access to food and water in the Veterinary Medical Unit in the VA Loma Linda Healthcare System. Mice were allowed to acclimate for one week before experiments were initiated. The animals were randomly assigned to groups that included anesthesia and TBI from heights of 0.5, 1.0, and 1.5 m. All experimental protocols were approved by the Institutional Animal Care and Use Committee of the VA Loma Linda Healthcare System. All procedures performed followed the ethical guidelines for animal studies.

### Traumatic brain injury

TBI was induced as previously reported.^19,20^ A 75 g brass weight was used to generate the impact on the skull. To obtain a consistent impact on the mouse skull, a tube was positioned above the mouse calvaria, between the ears. Under isoflurane anesthesia, mice were subjected to repeated mTBI from different heights (0.5, 1.0, or 1.5 m), once per day, for four consecutive days.^20^ Severity of TBI increased with drop height. The control mice received isoflurane anesthesia only. The righting response was recorded as previously described.^21^ All mice survived the duration of the experiment and no paralysis or skull fractures were observed. There were no weight differences between control [(17.30±0.07) g], 0.5 m [(17.30±0.13) g], 1.0 m [(16.53±0.10) g], and 1.5 m [(16.26±0.06) g] groups.

### Neuromotor assessment

Grip strength was used to measure the neuromuscular function of the fore and hind limbs in mTBI and in control mice.^22^ Briefly, mice were placed on a horizontal grid. The front and hind paws were at the same height as the grid, which was connected to a sensor. Once the optimal grip was established, the mice were pulled backward gently at a constant speed until the grip was released. Peak tension (grams of force) was recorded on a digital force (Newton) gauge as the mice released their grip. The transducer (Instrument) was set to 0 for each measurement. The tests were repeated in three successive trials while limb strength was measured at 2, 3, 4, 5 and 14 days post impact. Data was collected for each day and averaged.

### Serum analysis

Blood was collected from mice at day 5 and day 14 post impact using a retro-orbital method. The serum was collected and stored at −80 °C and used for quantitating IGF-I (R&D system, Minneapolis, MN, USA) and osteocalcin levels (ALPCO, Salem, NH, USA) with ELISA kits.

### Micro-computed tomography

For *ex vivo* and *in vivo *micro-computed tomography (μ-CT) evaluations, a high-resolution tomography image system (viva CT40; Scanco Medical, Switzerland) was used to measure cortical and trabecular bone parameters, as previously described.^23,24^ Routine calibration was performed once per week using a three-point calibration phantom corresponding to a density range from air to cortical bone. The bones were scanned at a resolution of 10 μm with a 55 and 65 kVp X-ray for trabecular and cortical bone. After acquiring the radiographic data, images were reconstructed using the 2-D image software provided by Scanco. Every 10 sections of the cortical or trabecular bone were outlined, and the intermediate sections were interpolated with the contouring algorithm to create a volume of interest, followed by the three dimensional analysis. For cortical bone measurements, 1 mm of cortical bone was scanned 3 mm away from the tibia–fibula junction (TFJ). We used the TFJ as a reference point to minimize variation arising from the bone position between mice. For trabecular bone measurements, the bones were scanned from 0.36 mm proximal to the growth plate. The exact numbers and location of the slices used for the analysis were corrected for bone length such that the analyzed regions were anatomically comparable between samples. Parameters such as bone volume (BV, mm^3^), bone volume fraction (BV/TV, %), apparent density (mg HA/ccm), trabecular number (Tb. N, mm^−^^1^), trabecular thickness (Tb. Th, μm) and trabecular space (Tb. Sp, μm) were evaluated in the impacted and non-impacted groups.

### Finite element analysis

We used finite element analysis (FEA) to determine whether changes in trabecular bone mass affected bone strength. Finite element models of the trabecular bone of tibial metaphysis were created directly from the segmented μ-CT aim objects (1-byte per voxel) using Image processing Language (IPL) software (FE-version 1.16) provided by Scanco, Switzerland.^25^ Scanco FE-software was used to directly convert voxels in the scanned volume of interest (VOI) into 8-node brick elements. Image voxels in the VOI were converted into approximately 127 070 elements at the tibial metaphysis. The Poisson’s ratio was set to 0.3 and Young Modules at 10 GPa. Standard boundary condition (number 33 from “scanco_fe_standard library”) was used to stimulate high friction compressive test in the *z* direction with 1% scale factor.^26^ The apparent modulus (N·mm^2^) which reflects strength based on the bone density was obtained from the fe_post file and was used to compare the difference in bone strength between TBI and control mice. The 1.5 m drop height impacted mice were chosen for comparison with the control mice since this group showed a significant reduction in trabecular bone mass as early as two weeks after the impact.

### Dynamic calcein labeling and histomorphometry

Mice were intraperitoneally injected with calcein (20 mg·kg^−1^) 10 and 4 days before euthanasia. Bone samples were collected and fixed in 10% formalin.^27^ Calcein labeling was visualized using the Olympus BX60 fluorescence microscope (Olympus, Center Valley, PA, USA) and analyzed using the OsteoMeasure software (Osteometrics, Decatur, GA, USA). Trabecular bone parameters such as mineral surface (MS, mm), bone formation rate (BFR, mm^2^×10^−3^ per day), and mineral apposition rate (MAR, μm per day) were calculated at the tibial metaphysis (secondary spongiosa) in the control and impacted mice as previously described.^28^ In addition, the bone resorbing surface was also measured by tartrate-resistant acid phosphatase (TRAP) staining at the trabecular site. The measurements were done at ×100 magnification.

### Statistical analysis

Regression analysis was used to test if there was a time dependent decrease in bone mass with the varying impact parameters in the separate impact groups. Analysis of variance (ANOVA) was used to compare differences between the groups for bone parameters, serum markers and grip testing. Student's *t*-tests were used to compare bone strength parameters. An alpha level of *P*<0.05 was considered statistically significant. Data was analyzed using IBM SPSS (version 21, Armonk, NY, USA) software and presented as the mean±s.e.m.

## Results

### Effect of repeated mTBI on skeletal parameters as a function of time

**Figure 1:**
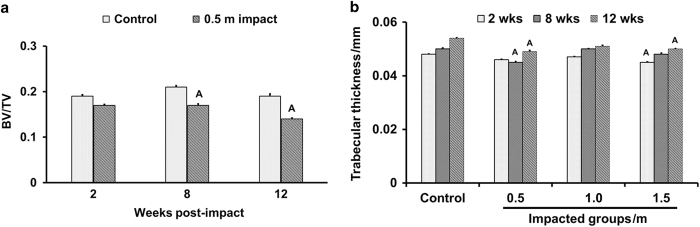
The time course effect of 0.5 m drop height impact on trabecular bone volume adjusted for tissue volume (**a**) and trabecular thickness (**b**). Values are mean±s.e.m. *n*=7–10. ^A^*P*<0.05 vs control.

Micro-CT analysis of trabecular bone at the secondary spongiosa revealed a 10%–26% reduction in trabecular BV/TV in impacted mice compared to control at different time points post impact (Table 1, Figure 1a). The 1.5 m drop height impacted mice showed a significant reduction in BV and BV/TV as early as 2 weeks post impact compared to other groups. At 8 weeks post impact, trabecular BV/TV was reduced by 10% and 19%, respectively, at 0.5 and 1.5 m impact heights compared to control mice (Table 1). At 12 weeks post impact, impacted mice showed a significant reduction in BV and BV/TV compared to control mice (Table 1). Consistent with the trabecular bone deficit in the tibia of impacted mice, the BV/TV in trabecular bone (L-5 vertebra) was also decreased by 5%–11% in three groups of impacted mice at 12 weeks post impact (Table 2). The reduced trabecular BV/TV in the TBI mice is mainly caused by changes in trabecular thickness (Figure 1a and b). ANOVA analysis revealed a time dependent reduction in the trabecular BV/TV in the 0.5 m drop height impacted mice (*P*=0.01) but not in the other two impacted groups.

We also measured cortical bone parameters at the diaphyseal site of the tibia in the impacted and control mice. We did not find significant changes in either BV or BV/TV parameters at 12 weeks post impact for any of the impacted groups compared to control mice (Table 3).

### Histomorphometric analysis

**Figure 2:**
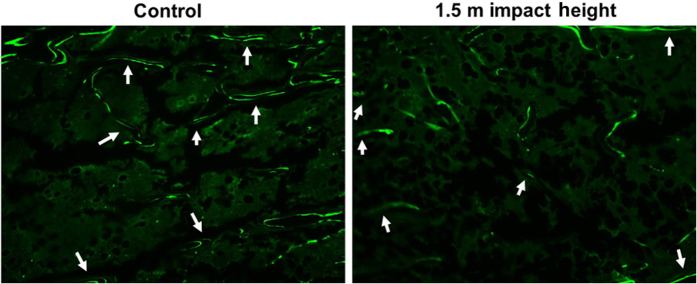
Longitudinal image of representative calcein labeled trabecular bone (secondary spongiosa) in 1.5 m drop height impacted and control mouse. Labeling (bright green) is representative of increased bone formation.

**Figure 3:**
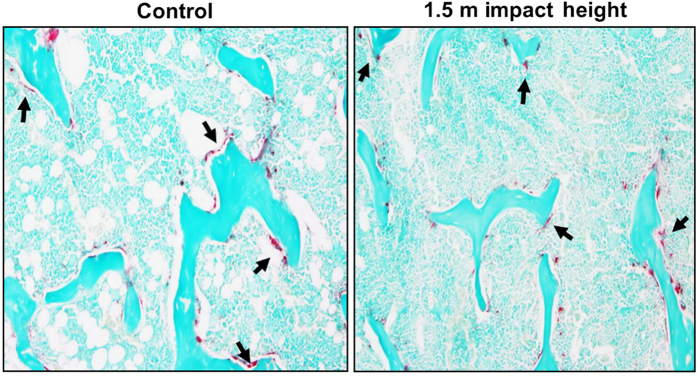
Representative image of tartrate-resistance acidic phosphatase (TRAP) labeled surface in the tibia metaphysis site (secondary spongiosa) of 1.5 m drop height impacted and control mouse.

Histomorphometric measurements at the secondary spongiosa of the tibia revealed that the bone formation rate was reduced by 55% (*P*<0.05) in the 1.5 m drop height impacted mice compared to control (Figure 2, Table 4). While the mineralizing surface and mineral apposition rate were reduced by 33% and 19%, respectively, these changes did not reach statistical significance. The resorbing surface measured by TRAP-labeled surface showed no significant difference in the 1.5 m drop height impacted mice compared to control (Figure 3, Table 4).

### Effect of repeated mTBI on bone strength parameters

**Figure 4:**
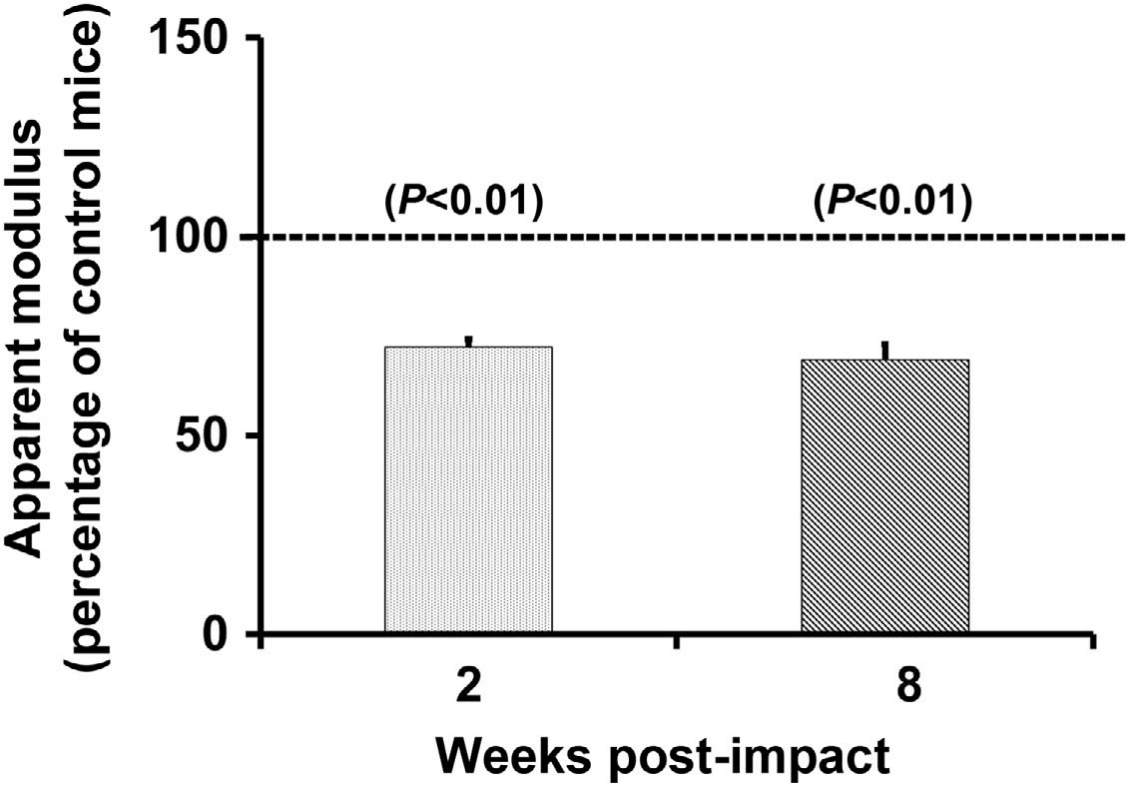
Decreased trabecular bone strength, reflected by apparent modulus in the 1.5 m drop height impacted mice compared to control at 2 and 8 weeks of post impact. Values are mean±s.e.m. *n*=7–10.

To determine if decreased trabecular bone mass in the TBI mice affected bone strength, the FEA program in μ-CT analysis was used to evaluate the trabecular bone strength in the impacted and control mice. We found that the apparent modulus was reduced by 30% (*P*<0.01) at the secondary spongiosa of the proximal tibia of the impacted mice compared to control mice at both 2 and 8 weeks post impact (Figure 4).

### Effect of repeated mTBI on serum IGF-I and osteocalcin levels

Serum IGF-I levels trended towards lower values in all three impacted groups compared to control mice at 5 days post impact, however, these changes did not reach statistical significance. At 14 days post impact, serum IGF-I levels were significantly less in the 0.5 m and 1.5 m drop height impacted groups compared to control (Table 5). There were no differences in serum osteocalcin levels between impacted mice and control at 5 days post impact. However, there was a significant reduction in serum osteocalcin at 0.5 m and 1.5 m impacts at 14 days post impact (Table 4). The 1.0 m drop height impacted mice showed a reduction in levels of IGF-I and osteocalcin but this reduction did not reach statistical significance (*P*=0.07).

### Relationship between serum parameters and trabecular bone mass

**Figure 5:**
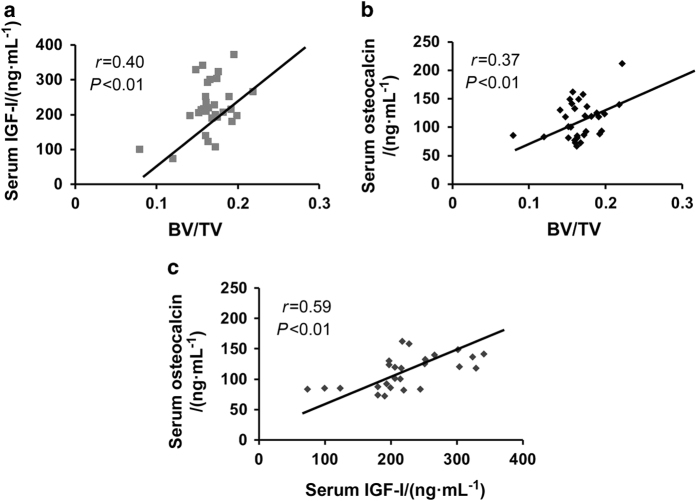
The significant positive relationships between (**a**) serum levels of IGF-I vs trabecular bone mass, (**b**) serum levels of osteocalcin vs trabecular bone mass and (**c**) serum levels of osteocalcin vs IGF-I in impacted and control mice l. *n*=31–36.

To test the relationship between decreased trabecular bone mass and changes in serum levels of osteocalcin and IGF-I, correlation analyses were performed. We found that trabecular BV/TV was positively correlated with serum IGF-I (*r*^*2*^=0.16, *P*<0.01) and osteocalcin (*r*^*2*^=0.14, *P*<0.01) levels, in both impacted and control mice (Figure 5). In addition, we found that the serum osteocalcin level showed a strong positive correlation with IGF-I in all mice (*r*^*2*^=0.35, *P*<0.01; Figure 5), suggesting that decreased IGF-I levels caused by repeated mild brain impacts could, in part, be responsible for the decreased trabecular bone mass.

### Neuromotor assessment

In order to confirm that the reduced trabecular bone mass in the TBI mice was not due to immobilization caused by impaired neuromotor function, we performed grip testing at 2, 3, 4, 5, and 14 days post impact. Multivariate analysis revealed no significant differences in the grip measurements between the impacted and control mice (Table 6). This finding suggests that the reduction in trabecular bone in impacted mice could not be attributable to reduced neuromotor function.

## Discussion

A considerable amount of research in the TBI field has been focused on neurocognitive and neuropathological changes and the risk of developing neurodegenerative diseases such as dementia after TBI. However, the long-term consequences of TBI in distal tissues such as the musculoskeletal system, has only been sparsely investigated. Our focus on TBI effects on the skeletal system was based on the establishment that skeletal growth and remodeling are under the control of neuroendocrine and neuronal signals that originate from the brain. To study the long-term effects of TBI on bone, we chose a weight drop model to induce injury for a number of reasons including: (1) it can be used to deliver repeated, mild injuries to the same animal, (2) the head and body are not constrained and the mouse falls freely upon impact and (3) the impact causes a very rapid and rotational acceleration of the head, an essential characteristic of blows to the head of humans exposed to concussive injuries. Furthermore, we have recently used this model to show that repeated mild TBI reduced trabecular bone mass acutely in mice.^20^ To further identify an optimal impact on the brain that produces consistent significant catabolic effects on bone, we subjected 10-week old female C57BL/6J mice to repeated mild TBI using a 75 g weight drop from different heights (0.5, 1.0 and 1.5 m), once per day for 4 consecutive days, on the skull. Since pathophysiological effects mediated by mTBI are both acute and long-term, we evaluated serum and skeletal parameters at different time points after impact from different heights. We found that repetitive mTBI significantly altered the trabecular bone microarchitecture and bone mechanical properties via endocrine dysfunction.

μ-CT analysis revealed a reduction in trabecular bone parameters in the impacted mice at different time points post impact (Table 1). Our data show that the 1.5 m drop height impacted mice exhibited a significant reduction in trabecular bone mass as early as 2 weeks post impact and that this reduction was maintained until the end of the study period (ie, 12 weeks post impact). By contrast, 0.5 and 1.0 m drop height impacted mice showed a significant reduction in trabecular bone mass at later time points (ie 8 and 12 weeks post impact). ANOVA analysis (*P*=0.01) revealed that there was a time dependent reduction in the trabecular bone volume adjusted for tissue volume in the 0.5 m drop height impacted mice. In addition, we did not detect significant correlations between the weeks post TBI and various drop heights. Overall, our findings seem to suggest that while all three impacts produced a catabolic effect on bone, brain injuries induced from a 1.5 m drop height produced greater and more rapid catabolic effects on bone than impacts from a 0.5 m drop height.

To determine the cellular processes contributing to the early reduction in trabecular bone mass in the 1.5 m impacted mice, we performed histomorphometric studies of bone formation and resorption parameters. We found that the bone formation rate at the trabecular site was reduced significantly in the 1.5 m drop height impacted mice. In terms of mechanisms for reduced bone formation, measures that reflect osteoblast number (mineralizing surface) and osteoblast activity (MAR) were reduced by 20%–30% but these changes were not significant. μ-CT analysis data revealed a significant reduction in trabecular thickness which reflects impaired osteoblastic activity in TBI mice. Accordingly, we found that the serum levels of osteocalcin, a measure of osteoblast function, were significantly reduced in the impacted mice compared to control. In contrast to bone formation, we found no significant differences in the TRAP labeled surface, a marker of osteoclast activity at the trabecular site in the impacted mice. In addition, our μ-CT analyses revealed no differences in trabecular separation, a marker of increased osteoclast activity. These data suggest that decreased bone formation in response to mTBI is the main cause of reduced trabecular bone mass in the impacted mice.

Assessment of bone quality using finite element analyses based on μ-CT images revealed a 30% a reduction in the apparent modulus in the 1.5 m drop height impacted mice compared to controls at 2 and 8 weeks post impact. This group was chosen for analysis because changes in trabecular bone mass occurred early and was maintained throughout the study period. Furthermore, the reduction in bone strength was similar at 2 and 8 weeks post impact suggesting that the decreased trabecular bone strength is an acute effect of brain injury that is maintained over a period of several weeks. Overall, our data suggests that repeated mTBI influences both trabecular architecture and strength that in turn, would lead to increased fragility and fracture risk.

Surprisingly, the experimental paradigm used in this study to generate mTBI produced a detrimental effect on trabecular bone but not cortical bone. There are a number of potential explanations for the differential effect of TBI on cortical versus trabecular bone. For example, it is known that the mechanisms and genetic control of cortical and trabecular bone formation are different. While much of the trabecular bone formation occurs via an endochondral bone formation route in which cartilaginous matrix formed by chondrocytes is subsequently replaced by bone matrix produced by osteoblasts, cortical bone formation occurs directly from osteoblasts-derived from periosteal bone cells. Furthermore, genetic loci that contribute to variation in peak bone density are known to be different for different skeletal sites.^29,30^ Thus, the TBI-induced changes in the neuroendocrine system in combination with neural signals may exert more profound effects on cells involved in endochondral bone formation than cortical bone formation. Alternatively, the lack of difference in cortical bone could be related to the site (3 mm away from tibia-fibular junction) used for measurement in this study.

**Figure 6:**
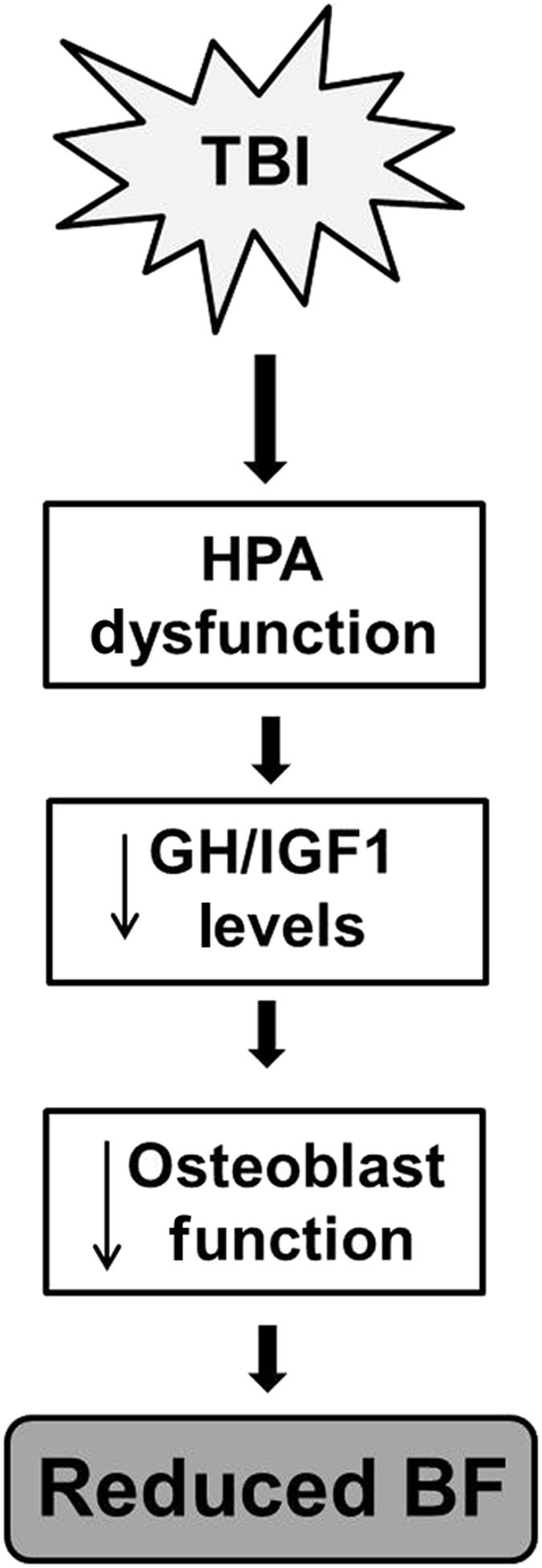
Diagram depicting the interplay between TBI and hormone dysfunction towards reduced bone formation.

To investigate the molecular mechanisms that contributed to the reduction in osteoblast function in mTBI mice, we focused on the GH-IGF-I axis. Reports have shown that patients with mild, moderate and severe TBI exhibit a high incidence of hypothalamus pituitary axis (HPA) dysfunction, which results in a temporary or permanent alterations of hormones secreted by the pituitary gland.^31^ In particular, reports have shown that GH deficiency is a common clinical manifestation of TBI and GH, a key regulator of skeletal growth and remodeling, mediates its anabolic effect on the skeleton either directly or indirectly through IGF-I. In this regard, mouse studies have shown that a lack of GH secretion resulted in a low level of IGF-I and that GH knock-out mice displayed osteopenia.^32,33^ Similarly, administration of IGF-I to GH deficient animals promoted skeletal growth.^34^ These data suggest that IGF-I plays an important role in mediating GH effects on the skeleton. Therefore, any imbalance in GH secretion should have a significant impact on the IGF-I levels and in turn, skeletal parameters. Since GH is secreted in a pulsatile manner, we measured IGF-I, a stable surrogate marker of GH, in the serum of impacted and control mice. We found that serum levels of IGF-I were decreased at 14 days post impact in all 3 groups of impacted mice. In addition, we found a positive correlation between serum IGF-I and trabecular bone volume, suggesting that GH/IGF-I deficiency caused by brain injury could in part contribute to the reduced trabecular bone mass (Figure 6). Serum levels of IGF-I correlated with osteocalcin, a marker of bone formation in the impacted and control mice. Further studies are needed to evaluate the cause and effect relationship between IGF-I reduction and bone formation changes in the mTBI mice.

Previous research has shown that small rodents with TBI exhibit motor deficits leading to immobilization and even signs of paralysis.^35^ Likewise, clinical studies also have shown that individuals with TBI exhibit difficulties in social communication and are less active due impaired motor function.^36–38^ Since motor impairments are common clinical manifestations of TBI, we investigated whether the decreased trabecular bone formation in the TBI mice was due to immobilization caused by disrupted motor function. We did not find significant differences in body weight or grip strength between impacted and control mice, suggesting that the decreased trabecular bone formation was not due to immobilization as a result of impaired motor function.

In conclusion, findings from our study demonstrate that (1) a 1.5 m drop height is effective in inducing a catabolic effect on trabecular bone mass and bone strength within 2 weeks post impact, (2) a low impact on the brain can have a severe catabolic effect on the bone over time and (3) mild TBI exerts its effects in part via by regulating GH/IGF-I-mediated bone formation. Future studies will address the relative contribution of deficiency in GH as well as other pituitary hormones to TBI-induced trabecula bone formation deficit.

The limitations of this study are as follows: First, while our study showed that repetitive mild TBI impacted trabecular bone mass in growing mice, it remains to be determined if TBI exerts similar effects in skeletally mature mice (4 months or older). Future studies using older mice (4, 12, and 18 months) are needed to determine whether the detrimental effects of TBI on the skeleton persist after skeletal maturity and in old age. Second, several studies have investigated the role of the peripheral nervous system in regulating bone formation.^39–41^ Nerve fibers of the sympathetic and sensory origin regularly innervate trabecular bone, periosteum, and fracture callus and are involved in the regulation of vascularization and matrix formation during endochondrial ossification.^42^ Furthermore, osteoprogenitors are known to originate from peripheral nerves and in patients with TBIs, fractures heal with callus formation at a higher rate.^43^ Accordingly, a recent study using a mouse model has shown that the altered function of peripheral sensory nerve resulted in decreased trabecular bone mass.^44^ Since mTBI mice also showed a decreased trabecular bone mass, future studies are necessary to determine if the decreased trabecular bone mass in mTBI mice is mediated in part due to altered sensory nerve function. Finally, we did not detect callus formation or ectopic bone in the mTBI mice. In this regard, it is possible that another insult (eg, injury) along with TBI may be needed to induce heterotopic ossification. Our future studies will address these and other mechanisms for the adverse effects of TBI on bone.

## Figures and Tables

**Table 1:** Micro-CT measurements of bone parameters at the metaphysis (secondary spongiosa) of the tibia in control and mTBI mice at 2, 8 and 12 weeks post impact

Bone parameters	Control	Weight drop height/m		
		0.5	1.0	1.5
*2 weeks post impact*				
BV/mm^3^	0.16±0.004	0.14±0.002	0.14±0.003	0.12±0.002^A^
BV/TV	0.19±0.003	0.17±0.002	0.17±0.003	0.16±0.003^A^
Tb.N/mm^−1^	6.11±0.09	5.86±0.05	5.85±0.07	5.71±0.06
Tb. Th/mm	0.048±0.000 2	0.046±0.000 2	0.047±0.000 2	0.045±0.000 2^A^
Tb. Sp/mm	0.17±0.002	0.17±0.001	0.16±0.003	0.18±0.001
Trabecular bone density (mg·cm^−3^)	164±1.93	142±3.0	151±2.29	139±1.06^A^
Cortical bone density (mg·cm^−3^)	587±1.24	586±2.68	594±2.29	575±1.34
				
*8 weeks post impact*				
BV/mm^3^	0.18±0.002	0.13±0.003^A^	0.17±0.001	0.15±0.002^A^
BV/TV	0.21±0.003	0.17±0.003^A^	0.22±0.002	0.19±0.003^B^
Tb.N/mm^−1^	5.79±0.05	5.53±0.03	5.83±0.04	5.48±0.01
Tb. Th/mm	0.05±0.000 4	0.04±0.000 4^A^	0.05±0.000 2	0.04±0.000 5
Tb. Sp/mm	0.16±0.001	0.17±0.001	0.16±0.001	0.17±0.005
Trabecular bone density (mg·cm^−3^)	179±2.30	153±2.82^A^	177±1.53	159±2.43^A^
Cortical bone density (mg·cm^−3^)	734±2.43	693±2.25^A^	697±2.75^A^	733±4.78
				
*12 weeks post impact*				
BV/mm^3^	0.15±0.000 3	0.10±0.002^A^	0.11 ±0.000 3^B^	0.11±0.002^A^
BV/TV	0.19±0.005	0.14±0.002^A^	0.16±0.003^B^	0.15±0.002^A^
Tb.N/mm^−1^	5.32±0.08	4.80±0.037^B^	4.90±0.043	4.89±0.03
Tb. Th/mm	0.054±0.000 2	0.04±0.000 5^A^	0.05±0.000 4	0.050±0.000 2^A^
Tb. Sp/mm	0.19±0.002	0.20±0.002	0.20±0.002	0.20±0.001
Trabecular bone density (mg·cm^−3^)	183±3.20	141±2.59^A^	152±2.69^A^	148±2.14^A^
Cortical bone density (mg·cm^−^^3^)	827±1.78	815±2.68	815±1.90	810±1.46^A^

Abbreviations: Micro-CT, micro-computed tomography; mTBI, mild traumatic brain injury.

Values are mean±s.e. *n*=7–10, ^A^*P*<0.05 vs control and ^B^*P*=0.07 vs control.

**Table 2:** Changes in trabecular parameters of the L5 vertebra 12 weeks post impact in control and mTBI mice

Bone parameters	Control	Weight drop height/m		
		0.5	1.0	1.5
BV/TV	0.34±0.003	0.30±0.002^A^	0.31±0.003^A^	0.31±0.001^A^
Tb.N/mm^−1^	5.34±0.07	5.01±0.06	4.95±0.07	4.88±0.03
Tb.Th/mm	0.065±0.002	0.062±0.000 4^B^	0.062±0.000 1^A^	0.062±0.000 1^A^
Tb.Sp/mm	0.20±0.003	0.21±0.002	0.21±0.004	0.22±0.002

Values are mean±s.e., *n*=9–10, ^A^*P*<0.05 vs control and ^B^*P*=0.07 vs control.

**Table 3:** Tibia cortical bone parameters 12 weeks post impact in the control and mTBI mice

Bone parameters	Controls	Weight drop height/m		
		0.5	1.0	1.5
TV/mm^3^	0.62±0.003	0.60±0.003	0.62±0.003	0.60±0.003
BV/mm^3^	0.38±0.002	0.36±0.003	0.39±0.003	0.37±0.001
BV/TV	0.61±0.000 8	0.61±0.001	0.62±0.001	0.61±0.001

Values are mean±s.e., *n*=10.

**Table 4:** Histomorphometric analysis of tibia at the metaphysis site (secondary spongiosa) in the control and 1.5 m impacted mice

Parameters	Control	Impacted	*P*-value
Mineralizing surface/mm	1.44±0.03	0.96±0.08	*P*=0.09
Bone formation rate (mm^2^×10^−3^ per day)	2.17±0.13	0.97±0.07	*P*<0.02
Mineral apposition rate (μm per day)	1.37±0.09	1.11±0.10	*P*=0.18
TRAP stained osteoclast surface/bone surface	9.83±0.45	11.86±0.49	*P*=0.30

Values are mean±s.e., *n*=7–8.

**Table 5:** Serum levels of IGF-I and osteocalcin in the control and mTBI mice at day 5 and 14 post impact

Marker	Day	Control	Weight drop height/m		
			0.5	1.0	1.5
IGF-I/(ng·mL^−1^)	5	242±6.89	233±7.22	227±10.04	218±6.18
	14	289±7.67	184±8.58^A^	223±6.72^B^	191±7.12^A^
Osteocalcin/(ng·mL^−1^)	5	128±3.89	139±3.09	131±5.13	144±4.0
	14	129±4.03	87±4.0^A^	98±4.29^B^	100±3.12^A^

Values are mean±s.e. *n*=7–10, ^*A*^*P*<0.05 vs control and ^B^*P*=0.07 vs control.

**Table 6:** Grip testing measured at different days after impact in the control and mTBI mice

Days post impact	Weight drop height/m			
	Control	0.5	1.0	1.5
2	1.74±0.013	1.68±0.018	1.62±0.005	1.56±0.009
3	1.80±0.011	1.77±0.025	1.75±0.012	1.71±0.015
4	1.76±0.012	1.79±0.017	1.76±0.017	1.60±0.011
5	1.79±0.015	1.75±0.017	1.69±0.013	1.60±0.012
14	1.95±0.009	2.01±0.013	1.91±0.015	1.80±0.015

Values are mean±s.e., *n*=7–10.
